# Case Report: Exome Sequencing Identified a Novel Frameshift Mutation of α*-Actin 1* in a Chinese Family With Macrothrombocytopenia and Mild Bleeding

**DOI:** 10.3389/fped.2021.679279

**Published:** 2021-06-18

**Authors:** Fang-Mei Luo, Liang-Liang Fan, Yue Sheng, Yi Dong, Lv Liu

**Affiliations:** ^1^Department of Respiratory Medicine, The Second Xiangya Hospital of Central South University, Changsha, China; ^2^Department of Cell Biology, The School of Life Sciences, Central South University, Changsha, China; ^3^Hunan Key Laboratory of Animal Models for Human Disease, School of Life Sciences, Central South University, Changsha, China

**Keywords:** macrothrombocytopenia, mild bleeding, ACTN1 mutation, non-sense-mediated mRNA decay, whole-exome sequencing

## Abstract

Inherited macrothrombocytopenia (IMTP) is a rare disorder characterized by a reduced platelet count and abnormally large platelets. The main clinical symptom of IMTP is mild bleeding in some patients. At present, more than 30 genes have been identified in patients with syndromic and non-syndromic IMTP. In this study, a 3-year-old boy and his mother who presented with mild epistaxis and/or gingival bleeding were diagnosed as having IMTP. Wen then selected whole sequencing to explore the genetic lesion of the patients. After data filtering and mutation validation, a novel frameshift mutation (NM_001130004: c.398_399insTGCG, p.F134AfsX60) of α*-actin 1* (*ACTN1*) was identified in the proband and his mother but absent in other unaffected individuals. Previous studies have proven that mutations in *ACTN1* may lead to IMTP with mild to absent bleeding phenotype. The novel mutation, resulting in a truncated protein in exon 4 of the *ACTN1* gene, was absent in the public database, such as 1000G and genomAD. Further Western blot revealed that the expression of α-actin 1 in the proband was decreased overtly, which indicated that the novel frameshift mutation may induce non-sense-mediated mRNA decay. In summary, this study not only broadened the variants spectrum of *ACTN1* gene, which may contribute to the genetic counseling of IMTP, but also confirmed the diagnosis of IMTP, which may help the management and prognosis for the family members.

## Introduction

Inherited macrothrombocytopenia (IMTP) is an important cause of thrombocytopenia, which is defined as a platelet count of <150 × 10^9^/L (1–3). Besides the reduced platelet count, a significant increase in platelet size (>12 fL) is another feature of IMTP (1, 3). As a rare clinical condition, IMTP affects at least 2.7 per 100,000 individuals with mild to absent bleeding phenotype (2, 4). At present, mutations in more than 10 genes including α*-actin 1*(*ACTN1*), *myosin heavy chain 9* (*MYH9*), *tubulin beta class I* (*TUBB*), etc. have been identified in non-syndromic IMTP with autosomal dominant, recessive, and sex-linked patterns (1, 3). In addition, some syndromes are also typically characterized by low platelet counts and severe bleeding tendency, such as Wiskott-Aldrich syndrome, Bernard-Soulier syndrome, Di George syndrome, and so on (5–7). However, because of varying mutations and clinical manifestations, the IMTP shows obvious heterogeneity, which challenges the clear diagnosis of IMTP and leads to the misdiagnosis as immune thrombocytopenic purpura (8).

In this study, we enrolled a 3-year-old boy and his mother with IMTP and mild epistaxis and/or gingival bleeding. The aim of this study was to explore the genetic lesion of the patients with the help of whole-exome sequencing.

## Case Presentation

**Figure 1:**
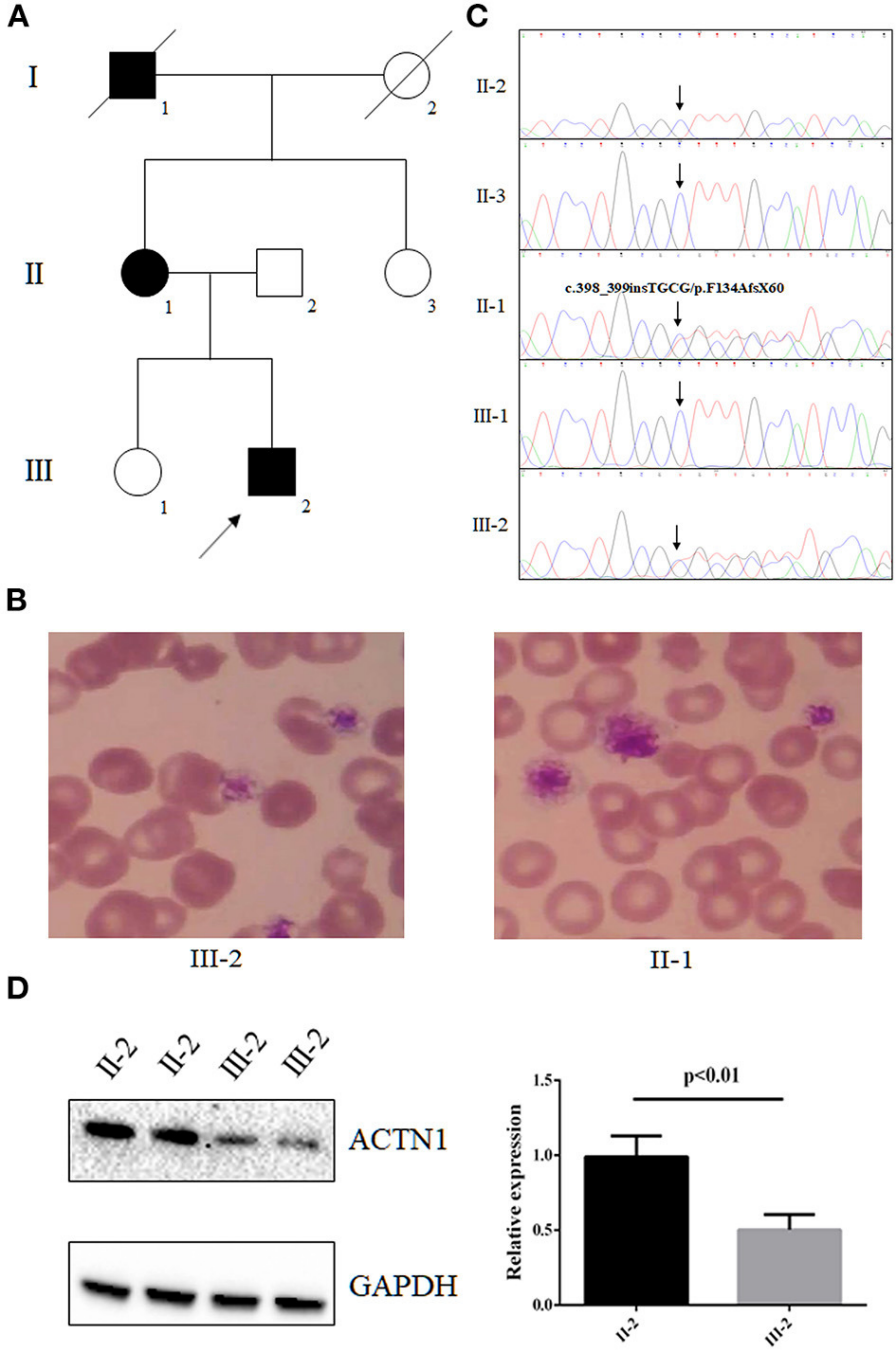
The clinical data and genetic analysis of the family with IMTP and mild bleeding. **(A)** Pedigree of the family. Black circles/squares are affected; white circles/squares are unaffected; slashed symbol is deceased family member. Arrow indicates the proband. **(B)** Blood smears (May-Grünwald Giemsa staining) revealed macrothrombocytopenia in the proband and his mother. **(C)** Sanger DNA sequencing chromatogram demonstrates the heterozygosity for an *ACTN1* frameshift mutation (NM_001130004: c.398_399insTGCG, p.F134AfsX60) in affected members. **(D)** Western blot analysis of the expression of ACTN1 in the proband's platelets.

A family from central south region of China (Hunan province) including seven persons was investigated in this study (Figure 1A). The proband (III-2), a 3-year-old boy, was admitted to our hospital because of mild epistaxis. Blood routine examination of the proband was shown as follows: hemoglobin, 13.0 g/dL; platelet count, 102 × 10^9^/L; mean platelet volume, 12.9 fL; and platelet distribution width, 18.1 fL. *In vitro* platelet aggregation in response to collagen and ristocetin was normal, but adenosine diphosphate (ADP) was slightly reduced (35%). Medical history survey found that the boy (III-2) has suffered from epistaxis several times with unexplained reason. Further family history investigation revealed that the proband's mother (II-1) and grandfather (I-1) have a history of mild epistaxis and gingival bleeding. Blood routine examination of the proband's mother (II-1) also found the reduced platelets (111 × 10^9^/L) count and increased mean platelet volume (12.7 fL) and platelet distribution width (17.7 fL). Peripheral blood smears May-Grünwald Giemsa staining revealed macrothrombocytopenia in the proband and his mother (Figure 1B). No other family members showed abnormal blood routine examination and bleeding diathesis. In addition, 200 unrelated, ethnically matched healthy controls were used as internal controls to exclude single-nucleotide polymorphisms (SNPs) in local individuals. These healthy controls (male/female: 100/100, aged 36.7 ± 8.6 years) lacked IMTP diagnostic features. Each participant underwent thorough examination for clinical diagnosis or exclusion, including general examination such as blood routine examination and peripheral blood smear May-Grünwald Giemsa staining.

We then employed whole-exome sequencing to explore the candidate gene mutation in the proband (III-2). Whole-exome sequencing was conducted at BerryGenomic Institute (Beijing, China) (9). Exomes were captured by Agilent SureSelect Human All Exon V6 kits, and high-throughput sequencing was conducted with an Illumina HiSeq 4000 system. The strategies of data filtering were as follows (9, 10): (a) non-synonymous SNPs or frameshift-causing INDELs with an alternative allele frequency >0.01 in the NHLBI Exome Sequencing Project Exome Variant Server (ESP6500), dbSNP155, the 1000 Genomes Project, the genomAD database, or in-house exome databases of BerryGenomic (2,000 exomes) were excluded; (b) the filtered SNVs and INDELs, predicted by SIFT, Polyphen2, and MutationTaster to be damaging, were remained; (c) the variants belong to pathogenic and likely pathogenic according to American College of Medical Genetics (ACMG) guideline remained (11); (d) cosegregation analysis was conducted in the family.

Whole-exome sequencing yielded 9.98-Gb data. After alignment and single-nucleotide variant calling, 70,145 variants were identified in the proband. Via the aforementioned filtering method and Sanger sequencing validation, a novel frameshift mutation (NM_001130004: c.398_399insTGCG, p.F134AfsX60) of *ACTN1* was identified in the proband and his mother but absent in unaffected individuals (Figure 1C). No other potential pathogenic mutation for macrothrombocytopenia-related phenotype was found (Table 1). Previous studies have proven that mutations in *ACTN1* may lead to IMTP with mild to absent bleeding phenotype (12, 13). The novel mutation, resulting in a truncated protein in exon 4 of the *ACTN1* gene, was absent in the public database such as 1000G and genomAD, as well as our 200 healthy controls. Bioinformatics programs predicted that this mutation (NM_001130004: c.398_399insTGCG, p.F134AfsX60) was a pathogenic mutation and located in an evolutionarily conserved site of the α-actin 1 protein. According to ACMG guidelines (11), this mutation was pathogenic (PVS1 + PM2 + PM3). We then further extracted total protein from the platelet of the proband and healthy control (II-2). Western Blot indicated that, compared with the healthy controls, the expression of *ACTN1* was decreased by ~49% in the heterozygous carrier (Figure 1D). These data suggested that the variant (NM_001130004: c.398_399insTGCG, p.F134AfsX60) of *ACTN1* was a loss-of-function mutation and can lead to non–sense-mediated mRNA decay.

**Table 1:** Characteristics of the genetic variants identified for the proband via whole-exome sequencing.

**Gene**	**Transcript**	**cDNA**	**Protein**	**Genotype**	**dbSNP**	**ClinVar**	**ACMG level**	**OMIM phenotype**
CACNB2	NM_201590.2	c.1508C>T	p.S503L	het	rs137886839	Conflicting	Likely pathogenic	Brugada syndrome 4
LIMA1	NM_001113546.1	c.73C>A	p.L25I	het	rs140372565	Association	Likely pathogenic	[Low-density lipoprotein cholesterol level QTL 8]
PNPO	NM_018129.3	c.148G>A	p.E50K	het	rs549477447	Uncertain significance	Likely pathogenic	AR: pyridoxamine 5′-Phosphate oxidase deficiency
COL7A1	NM_000094.3	c.2392G>A	p.G798R	het	rs202237834	Uncertain significance	Likely pathogenic	AD,AR: epidermolysis bullosa dystrophica
IL17RD	NM_017563.4	c.572C>T	p.P191L	het	rs200088377	Likely pathogenic	Likely pathogenic	AD, AR: hypogonadotropic hypogonadism
ARL13B	NM_182896.2	c.568A>G	p.I190V	het	rs193219215	—	Likely pathogenic	AR: Joubert syndrome
C9	NM_001737.4	c.346C>T	p.R116*	het	rs121909592	Pathogenic	Likely pathogenic	C9 deficiency
DNAH11	NM_001277115.1	c.11804C>T	p.P3935L	het	rs72658814	Uncertain significance	Likely pathogenic	AR: ciliary dyskinesia, primary
KCNH2	NM_000238.3	c.2771G>C	p.G924A	het	rs199473009	Uncertain significance	Likely pathogenic	AD: long QT syndrome 2
**ACTN1**	**NM_001130004.1**	**c.398_399insTGCG**	**p.F134AfsX60**	**het**	**—**	**—**	**Pathogenic**	**AD: bleeding disorder, platelet type**
FANCL	NM_018062.3	c.738dup	p.M247YfsX4	het	**—**	**—**	Likely pathogenic	AR: Fanconi anemia

*AD, autosomal dominant; AR, autosomal recessive. The MAF of these mutations was <0.01 in ESP6500, dbSNP155, the 1000 Genomes Project, the genomAD database, and in-house exome databases of BerryGenomic*.

*The bold words represent the genetic mutation which was responsible for this family*.

## Discussion

The human *ACTN1* gene encoding a member of the actin-crosslinking protein named α-actinin is located on chromosome 14q24.1, and it consists of 21 exons spanning ~3.78 kilobases. α-Actin 1 participates in the organization of the cytoskeleton, thought to be an anchor actin to a variety of intracellular structures and mainly expressed in platelets and megakaryocytes (14). In 2013, six different mutations of *ACTN1* were identified in 13 unrelated families with IMTP, which indicated that *ACTN1* was one of the genetic lesions in IMTP (12). At present, thrombocytopenia caused by pathogenic variants in *ACTN1* gene has been classified to ACTN1-related thrombocytopenia (15). To date, approximately 44 mutations of *ACTN1* have been detected in IMTP patients. Here, we identified a frameshift mutation (NM_001130004: c.398_399insTGCG, p.F134AfsX60) of *ACTN1* in a Chinese family with IMTP. As far as we know, this mutation may be first reported in IMTP patients; our study expanded the variant spectrum of *ACTN1* gene.

The α-actin superfamily consists of four members including ACTN1, ACTN2, ACTN3, and ACTN4, which are responsible for the organization of the cytoskeleton (3, 16). Previous studies have found that *ACTN1* was mainly expressed in platelets and megakaryocytes; the mutated *ACTN1* may lead to a decrease of 50% platelet counts and an increase of 30% in platelet size (12, 17). In our study, the proband and his mother with the novel mutation of *ACTN1* were also presented with macrothrombocytopenia. The novel frameshift mutation (NM_001130004: c.398_399insTGCG, p.F134AfsX60) can lead to the truncation mutation in the N-terminal of ACTN1. According to non–sense-mediated mRNA decay theory (18), the novel mutation may result in the decreased mRNA levels of *ACTN1*. Functional studies further confirmed that the novel mutation may lead to the reduction of *ACTN1* expression, which may affect the organization of the cytoskeleton in platelets and megakaryocytes, finally resulting in macrothrombocytopenia.

Previous studies in Chinese hamster ovary cells revealed that the mutated *ACTN1* may disrupt the normal actin-based cytoskeletal structure (12). The mice with *ACTN1* mutation may present with disorganized actin-based cytoskeleton in megakaryocytes, which may further result in abnormal number and size of platelets (12). In this study, the phenotypes of the novel mutation (NM_001130004: c.398_399insTGCG, p.F134AfsX60) carriers (II-1 and III-2) were consistent with mice model and other reported patients, which further confirmed the pathogenicity of this novel mutation (19, 20).

IMTP can easily be misdiagnosed as immune thrombocytopenic purpura, which may further make problems in therapy and management of patients (8). Hence, precise diagnosis is necessary for IMTP patients, especially for the IMTP patients caused by *ACTN1* mutations. A recent study that involved ~50 *ACTN1* mutation carriers indicated that *ACTN1* mutations lead to a benign form of platelet macrocytosis not always associated with thrombocytopenia (21). Precise diagnosis of *ACTN*1-caused IMTP can provide affected patients and their family members with a good prognosis. In our study, we confirmed the diagnosis of the affected members by whole-exome sequencing and Sanger sequencing, which may aid in the further management and prognosis of the family members. Meanwhile, our study also indicated that whole-exome sequencing combined with Sanger sequencing was an effective method in diagnosis of IMTP.

In summary, by employing whole-exome sequencing, we identified a novel frameshift mutation (NM_001130004: c.398_399insTGCG, p.F134AfsX60) of *ACTN1* in a Chinese family with IMTP and mild epistaxis and/or gingival bleeding. Hence, this study not only broadened the variants spectrum of *ACTN1* gene, which may contribute to the genetic counseling of IMTP, but also confirmed the diagnosis of IMTP, which may help in the management and prognosis of the family members.

## Data Availability Statement

The data presented in the study are deposited in the (BioSample) repository, accession number: SAMN18953804, https://www.ncbi.nlm.nih.gov/biosample/18953804.

## Ethics Statement

The studies involving human participants were reviewed and approved by the Second Xiangya Hospital of Central South University, Changsha, China. Written informed consent to participate in this study was provided by the participants' legal guardian/next of kin.

## Author Contributions

F-ML and LL enrolled the samples and clinical data. YS and YD performed DNA isolation and PCR. F-ML and L-LF wrote the manuscript and revised it. L-LF and LL supported the project. All authors reviewed the manuscript.

## Conflict of Interest

The authors declare that the research was conducted in the absence of any commercial or financial relationships that could be construed as a potential conflict of interest.
