# Supplementary figures and images for: Case Report: Exome Sequencing Identified a Novel Frameshift Mutation of α-Actin 1 in a Chinese Family With Macrothrombocytopenia and Mild Bleeding
Source: Front Pediatr. 2021 Jun 18;9:679279. doi: 10.3389/fped.2021.679279 (PMC8253534; doi:10.3389/fped.2021.679279)

（1）the original gel(s) of Western Blot in this study.


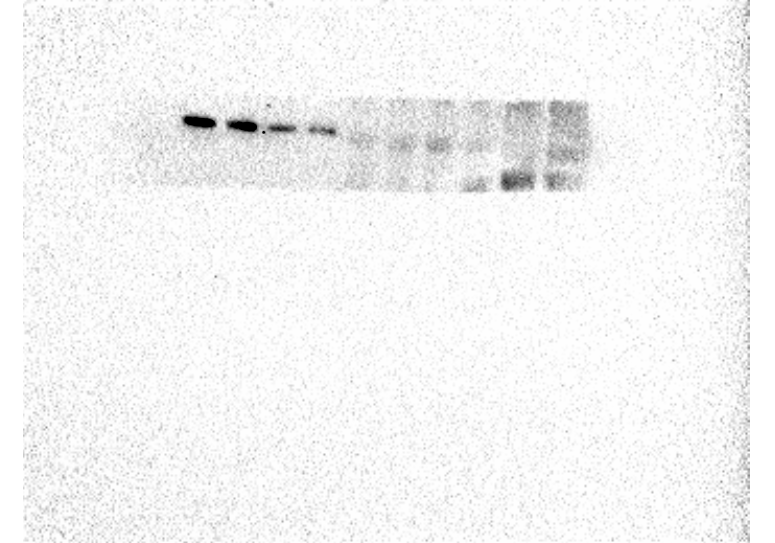


ACTN1


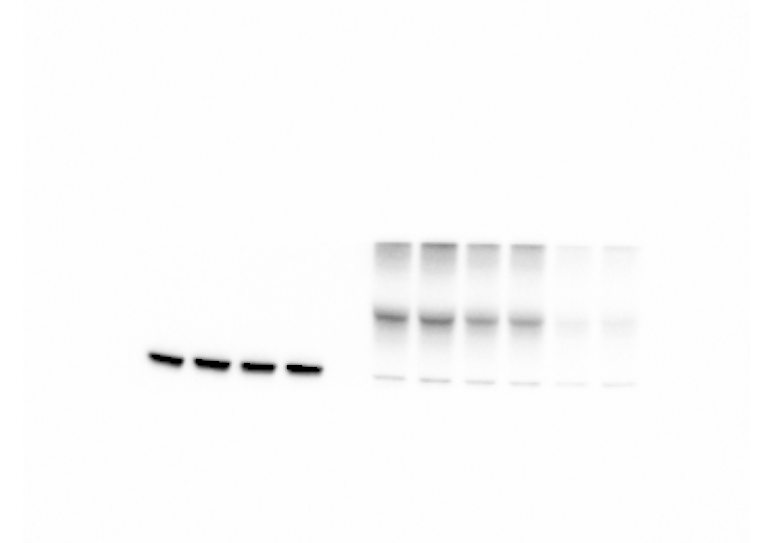


GAPDH

(2) The reverse sequence of the proband.


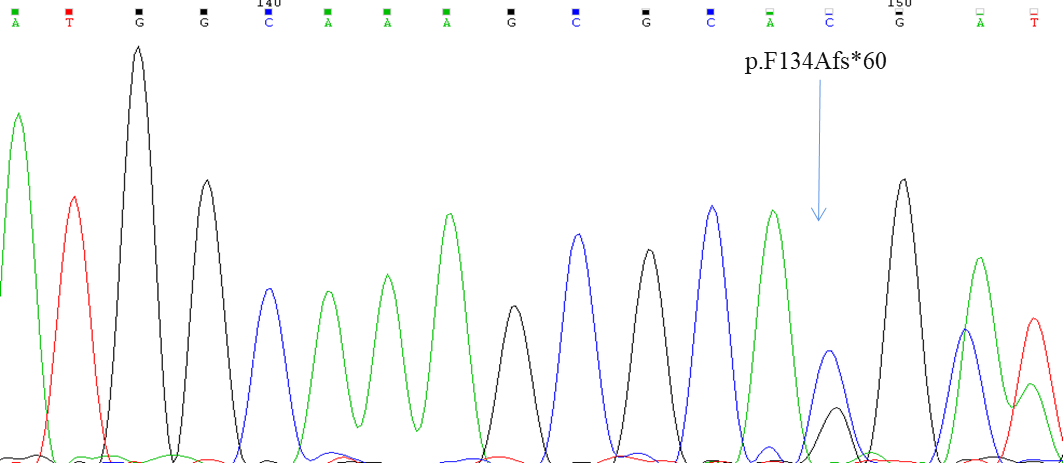

Supplement: Supplementary file 1 [file Data_Sheet_1.DOCX]
